# Increased risk of second primary malignancies following uterine cancer: a population-based study in Taiwan over a 30-year period

**DOI:** 10.1186/s12885-015-1426-3

**Published:** 2015-05-11

**Authors:** Kuan-Der Lee, Chao-Yu Chen, Huei-Jean Huang, Ting-Yao Wang, David Teng, Shih-Hao Huang, Chyong-Huey Lai, Min-Chi Chen

**Affiliations:** 1 Department of Hematology and Oncology, Chang Gung Memorial Hospital at Chiayi and Chang Gung University College of Medicine, Taoyuan, Taiwan; 2 Department of Obstetrics and Gynecology, Chang Gung Memorial Hospital at Linkou and Chang Gung University College of Medicine, Taoyuan, Taiwan; 3 Department of Bioengineering, University of California, Los Angeles, USA; 4 Biostatistics Consulting Center and Department of Public Health, College of Medicine, Chang Gung University, Taoyuan, Taiwan

**Keywords:** Uterine cancer, Second primary malignancy, Standardized incidence ratios

## Abstract

**Background:**

Previous studies assessing second primary malignancies (SPMs) after uterine cancer have been conducted in Western populations with conflicting results. This study aimed to define the incidence and risk of SPMs in Taiwanese patients with an initial diagnosis of uterine cancer.

**Methods:**

Using population-based data from the Taiwan Cancer Registry for the period 1979–2008, we quantified standardized incidence ratios (SIRs) among 11,571 women with an initial diagnosis of uterine cancer.

**Results:**

Among the 11,571 women, 555 (4.80 %) developed at least one SPM during 69,987 person-years of follow-up. There was a 71 % increased risk of SPM following uterine cancer (SIR = 1.71, 95 % CI, 1.57–1.86), with higher risks in the vagina/vulva (SIR = 9.06), small intestine (SIR = 8.45), ovary (SIR = 4.15), urinary bladder (SIR = 2.31), kidney (SIR = 2.24), colorectum (SIR = 2.24), lung (SIR = 1.96), and breast (SIR = 1.43). The risk of SPM was found to be the highest within the first 5 years after diagnosis of uterine cancer, with surveillance bias possibly contributing to the extremely high risk observed in the first follow-up year. The overall risk and pattern of SPM development observed in this study differed from those previously reported in Western populations, possibly because of the methodology and shorter follow-up period employed in this study. The cumulative incidence of SPMs was significantly higher in older patients (≥50 years) than in younger patients (P < 0.001).

**Conclusions:**

To our knowledge, this is the first study in an Asian population to report 71 % increased risk in SPMs in women previously diagnosed with uterine cancer. A younger age at diagnosis of uterine cancer conferred an increased risk of second malignancies, and SPMs worsened survivorship in patients who survived uterine cancer.

## Background

Uterine cancer is the sixth most common malignancy in women worldwide [1]. In Western countries, it is the most common gynecological malignancy, with a continuously rising incidence rate [1-3]. For example, the incidence of endometrial cancer reportedly increased by 21 % from 2008–2012 in the United States [3]. The incidence in Asian countries has been lower but has markedly increased in recent decades [4, 5]. In Taiwan, the incidence of uterine cancer has more rapidly increased than other malignancies from 3.43–15.07 per 100,000 between 1996 and 2010 [6, 7]. It is noteworthy that age at onset of uterine cancer among Asian patients has been reported to peak at 45–55 years, at least 10–15 years earlier than that reported in Western patients [4]. A similar trend in age at breast cancer diagnosis has been reported in Asian patients [8]; however, the mechanisms underlying the lower age at onset for both cancers remain unclear. Further evaluation of the risk of second primary malignancies (SPMs) following uterine cancer may provide some insights to its etiology.

Similar to the United States, uterine cancers in Taiwan comprise ~90 % adenocarcinomas, 8 % sarcomas, and other unspecified types [9, 10]. The 5-year overall survival rate for uterine cancers recorded in the Surveillance, Epidemiology, and End Results (SEER) database (1988–2001) was found to be 87.9 %. Of all patients in this database, 75.3 % had stage I disease, and these patients had a five-year survival rate of 97.4 % [9]. Considering the increasing incidence and survival rate of patients with uterine cancer worldwide, SPMs have become an increasingly important clinical issue. Nevertheless, most studies about SPMs after uterine cancer have been conducted in Western populations [11-18]. The most comprehensive evaluation of the SEER database has demonstrated markedly elevated SIRs for several specific sites; however, the overall SIR was not found to be increased [19]. Similarly, two large SEER studies based on 90,502 and 98,205 patients with primary endometrial cancer from 1973–2004 and 1973–2007, respectively, revealed that the overall risk of SPMs did not differ from the general population [14, 18]. In contrast, a nationwide Swedish population-based study reported a 54 % increased risk in overall SPMs [13], and other studies have reported a higher risk of developing SPMs after uterine cancers in patients with a young age at diagnosis [17], African ancestry [18], Lynch syndrome (LS), family history of breast or ovarian cancer [12, 13, 17], and in those receiving radiotherapy [15, 16].

There are racial disparities in the SEER database [18]. The overall risk of SPMs after endometrial cancer was found to be higher in Americans of an African descent (SIR = 1.19; 95 % CI, 1.08–1.31) and lower in Caucasians (SIR = 0.85; 95 % CI, 0.84–0.87) compared with the general population. Furthermore, the pattern of SPMs was found to differ between African American and Caucasian populations. Therefore, there is a clinical requirement for studies assessing SPMs following uterine cancers in Asian populations, which, to our knowledge, have not been previously reported. To achieve this objective, we conducted a retrospective population-based study using a database from the Taiwan Cancer Registry (TCR) that included a total of 12,509 patients with an initial diagnosis of uterine cancer between 1979 and 2008.

## Methods

### Data sources

We quantified the incidence of SPMs among the 12,509 patients with an initial diagnosis of uterine cancer (International Classification of Diseases, 9th Revision (ICD-9) codes: 179 and 182) reported in TCR (http://crs.cph.ntu.edu.tw/) from January 1, 1979–December 31, 2008. This TCR was founded in 1979 and is financed by the Ministry of Health and Welfare for estimating the incidence of cancer in Taiwan. It is a population-based cancer registry that covered 22 million people in 2003. Furthermore, the hospitals with more than 50 beds are required to submit information regarding patients with newly diagnosed cancer to TCR, which reimburses the hospitals based on the reported number of cases for reducing the possibility of under-reporting. All cancer registry databases in TCR have been systemically converted to ICD-9 codes [20] and linked with death certificates from the national death database. Therefore, individuals not identified by this process were considered to be alive for the purpose of the current study (passive follow-up). Coding of multiple primaries followed the principles of International Association of Cancer Registries (IACR) and International Agency for Research on Cancer (IARC) [21-23]. Informed consent was not required because all registry records are anonymous and accessible to the public. The study was approved by the Ethics Committee of the Chang Gung Memorial Hospital.

Since 1996, 80 %–90 % of patients with uterine cancer have received hysterectomy as the standard treatment in Taiwan, limiting the risk of secondary cervical cancer [6, 7]. One hundred and three patients with a second cervical cancer (ICD-9: 180) were excluded from the analysis as discrimination between cervical cancer, endometrial cancer extending to the cervix, and cervical recurrence was challenging in patients who did not receive hysterectomy. Thus, this study evaluated the risk of secondary non-cervical cancers. We aimed to accurately determine the age at onset, estimate the person-years of follow-up, and minimize potentially unconfirmed cancer diagnoses in this study cohort. A total of 835 patients were excluded from analysis because they met one or more of the following criteria: (1) missing birth date (7 cases); (2) missing follow-up date or death status (128 cases); (3) SPM diagnosis or death occurring less than 1 month after diagnosis of uterine cancer (684 cases); or (4) age under 20 years (18 cases). Thus, 11,571 patients were included in the analysis.

### Statistical analysis

SIRs and the corresponding 95 % CIs were calculated [24] for all types of SPMs except for uterine corpus to quantify the rate of second malignancy development after diagnosis of uterine cancer. SIRs were calculated as the ratio of the observed number (O) of SPMs to the expected number (E), assuming the patients had the same incidence of cancer as the general female population. The number of person-years at risk was defined as the number of years from the date of diagnosis of uterine cancer to the date of death, date of SPM diagnosis, or end of the study period (December 31, 2008), whichever occurred first. The person-years of observation for each 5-year age group, 5-year period (1979–1983, 1984–1988, 1989–1993, 1994–1998, 1999–2003, and 2004–2008), and the time from entry to the cohort (≤1, 1–5, 5–10, or >10 years) were multiplied by cancer incidence rates for the Taiwanese female population. The corresponding products were summed over all ages and calendar years to yield the expected numbers of SPMs at each site. SIR confidence intervals were based on an assumed Poisson distribution of SPM cases. An approximate *χ*^2^ test was used for evaluating differences between two SIRs and trends in SIRs.

Survival curves of patients with uterine cancer diagnosed at age <50 and ≥50 years were calculated using the Kaplan–Meier method. Differences between the two age groups were examined by the log-rank test. Further, the Cox model with a time-dependent covariate [25, 26], allocating follow-up time for each patient to the non-SPM group until SPM occurrence, was used for comparing the survival between patients with and without SPM. All statistical tests were two-sided, and a P value of less than 0.05 was considered statistically significant.

## Results

### Patient characteristics

Of the 11,571 women with an initial diagnosis of uterine cancer (4,445 diagnosed at age <50 years and 7,126 at age ≥50 years) and the complete data available for analysis, 555 (4.80 %) developed SPM during 69,987 person-years of follow-up (Table 1). Of all the enrolled patients, 10.47 % were diagnosed with uterine sarcoma, and the remaining with endometrial cancer. Overall, uterine cancer was diagnosed at a mean age of 52.93 years, and the mean age at SPM diagnosis was 61.23 years. The average follow-up time was 6.05 years, including 9,638 cases (83 %) followed up for at least one year, 2,678 (23 %) for 5–10 years, and 2,518 (22 %) for >10 years. The average interval between the first and second cancers was 6.27 years, with a standard deviation of 5.53 years.

**Table 1:** Characteristics of population-based cohort of 11,571 patients first diagnosed as uterine cancer (ICD-9: 179, 182) in Taiwan 1979-2008

	All	<50 years old^a^	≥50 years old^a^
No. with uterine cancer	11,571	4,445 (38.42 %)	7,126 (61.58 %)
No. who developed a SPM (%)	555 (4.80 %)	163 (3.67 %)	392 (5.50 %)
Average age at diagnosis of uterine cancer ± SD (years)	52.93 ± 11.69	41.63 ± 6.22	59.98 ± 8.26
Average age at diagnosis of SPM ± SD (years)	61.23 ± 11.78	48.59 ± 8.57	66.50 ± 8.49
Average interval between first and second cancers ± SD (years)	6.27 ± 5.53	6.61 ± 5.68	6.14 ± 5.47
Average follow-up (years)	6.05 ± 5.55	7.00 ± 5.84	5.46 ± 5.27

*SD* standard deviation, *SPM* second primary malignancy

^a^Age at uterine cancer diagnosis. An age of 50 years was selected for comparison as this is the average menopausal age in Taiwanese women

### Risk of second primary malignancies stratified by site

SIRs and the corresponding 95 % CIs for SPMs at all sites, except the uterine corpus, were calculated. Patient ages at initial uterine cancer and SPM diagnoses are presented in Table 2. Irrespective of the site, the overall SIR for developing SPM was 1.71 (95 % CI, 1.57–1.86). Regarding SPM sites, the risk of developing genital malignancies, including ovarian, vaginal, and vulvar, was the highest (SIR = 4.91; 95 % CI, 3.71–6.37), followed by the urinary system (SIR = 2.27; 95 % CI, 1.64–3.06) and digestive system (SIR = 1.71; 95 % CI, 1.49–1.96). In particular, there was an increased risk of vaginal/vulvar (SIR = 9.06), small intestinal (SIR = 8.45), ovarian (SIR = 4.15), urinary bladder (SIR = 2.31), kidney (SIR = 2.24), colorectal (SIR = 2.24), lung (SIR = 1.96), and breast (SIR = 1.43) cancers (Table 2).

**Table 2:** Observed (O) and expected (E) numbers of second primary cancers after diagnosis of an initial uterine cancer (ICD-9 179, 182) in Taiwan 1979-2008

SPM site (ICD-9 code)	Age of uterine cancer (years) (mean ± SD)	Age of SPM (years) (mean ± SD)	O	E	SIR (O/E)	(95 % CI)
**Head and neck**	57.89 ± 11.95	63.67 ± 12.94	9	10.51	0.86	(0.39, 1.63)
Oral & pharynx (141, 143-5, 146,148-9)	49.50 ± 5.92	54.25 ± 8.96	4	4.81	0.83	(0.22, 2,13)
Nasopharynx (147)	71.67 ± 8.33	77.00 ± 6.93	3	4.48	0.67	(0.13, 1.96)
Larynx (161)	57	70	1	0.41	2.45	(0.03, 13.64)
Major salivary glands (142)	51	55	1	0.80	1.24	(0.02, 6.92)
**Digestive system**	56.21 ± 10.12	63.28 ± 10.41	212	124.06	**1.71**	(1.49, 1.96)
Esophagus (150)	58.00 ± 1.41	65.50 ± 7.78	2	1.57	1.28	(0.14, 4.61)
Stomach (151)	59.92 ± 9.06	66.88 ± 8.59	25	17.09	1.46	(0.95, 2.16)
Small intestine (152)	50.33 ± 10.45	56.50 ± 11.34	12	1.42	**8.45**	(4.36, 14.77)
Colorectum (153,154)	54.70 ± 10.43	61.22 ± 10.42	119	53.05	**2.24**	(1.86, 2.68)
Liver (155)	60.16 ± 6.81	69.09 ± 6.72	45	39.42	1.14	(0.83, 1.53)
Biliary system (156)	63.67 ± 15.95	71.67 ± 13.43	3	4.82	0.62	(0.13, 1.82)
Pancreas (157)	48.50 ± 12.57	54.17 ± 12.98	6	6.69	0.90	(0.33, 1.95)
**Genital system**	49.48 ± 12.52	52.57 ± 13.82	56	11.42	**4.91**	(3.71,6.37)
Ovary (183)	48.40 ± 11.54	49.75 ± 11.43	40	9.65	**4.15**	(2.95, 5.64)
Vagina and vulva (184)	52.19 ± 14.75	59.63 ± 16.92	16	1.77	**9.06**	(5.17, 14.71)
**Urinary system**	55.95 ± 13.10	64.14 ± 12.18	43	18.94	**2.27**	(1.64, 3.06)
Urinary bladder (188)	53.89 ± 15.02	62.28 ± 14.42	18	7.79	**2.31**	(1.37, 3.65)
Kidney (189)	57.44 ± 11.62	65.48 ± 10.38	25	11.16	**2.24**	(1.45, 3.31)
Lung and bronchus (162)	58.63 ± 10.19	64.97 ± 10.99	72	36.81	**1.96**	(1.53, 2.46)
Sarcoma (171)	48.50 ± 16.81	52.50 ± 16.45	6	6.77	0.89	(0.32, 1.93)
Skin (173)	52.50 ± 11.57	59.14 ± 12.38	14	11.85	1.18	(0.65, 1.98)
Breast (174)	53.41 ± 10.20	59.23 ± 10.46	95	66.51	**1.43**	(1.16, 1.75)
Brain (191)	52.00 ± 9.02	54.75 ± 9.25	4	2.25	1.78	(0.48, 4.55)
Thyroid (193)	55.46 ± 10.61	59.77 ± 11.50	13	9.84	1.32	(0.70, 2.26)
Leukemia (204-8)	51.33 ± 7.12	58.00 ± 9.80	9	5.07	1.77	(0.81, 3.37)
Lymphoma (200-3)	61.00 ± 7.62	67.57 ± 6.58	7	8.39	0.83	(0.33, 1.72)
Others	50.27 ± 13.00	56.93 ± 13.80	15	12.54	1.20	(0.67, 1.97)
**Total**	55.01 ± 11.45	60.54 ± 12.08	555	324.95	**1.71**	(1.57, 1.86)

Bold denotes statistical significance

*SD* standard deviation, *SIR* standardized incidence ratio, *SPM* second primary malignancy, *O* observed numbers of SPMs, *E* expected numbers of SPMs, *CI* confidence interval

### Risk of second primary malignancies stratified by age at diagnosis of uterine cancer

For the eight sites with an increased risk of SPMs, SIRs were further stratified according to age at diagnosis of uterine cancer (<50 and ≥50 years, Table 3, left half). Overall, patients <50 years at onset had a slightly higher risk of developing SPM than those aged ≥50 years (SIR = 2.44 vs. 1.98, P = 0.06). The age-based trend was the most prominent for small intestinal, colorectal, and ovarian SPMs (P = 0.005, 0.001, and 0.018, respectively), whereas the risk of lung, breast, vaginal, urinary bladder, and kidney SPMs was similar between age groups.

**Table 3:** Risk for significant second primary cancers by age at diagnosis of uterine cancer (left half) and follow-up interval (right half), respectively

SPM site (ICD-9 code)	Age^a^ (years)	O	E	SIR (O/E)	95 % CI	Interval^b^ (years)	O	E	SIR (O/E)	95 % CI
Small intestine (152)	<50	7	0.27	**26.06**	(10.44, 53.71)	≤5	6	0.23	**25.95**	(9.48-56.49)
	≥50	5	1.15	**4.34**	(1.40, 10.14)	≤1	0	0.03	**0**	NA
						1-5	6	0.20	**29.47**	(10.76-64.15)
						5-10	3	0.38	**7.97**	(1.60-23.30)
						≥10	3	0.81	3.69	(0.74-10.79)
Colorectum (153,154)	<50	36	9.68	**3.72**	(2.60, 5.15)	≤5	58	9.00	**6.45**	(4.89-8.33)
	≥50	83	43.36	**1.91**	(1.52, 2.37)	≤1	12	1.07	**11.25**	(5.81-19.66)
						1-5	46	7.93	**5.8**	(4.25-7.74)
						5-10	29	14.05	**2.06**	(1.38-2.96)
						≥10	32	30.00	1.07	(0.73-1.51)
Lung (162)	<50	13	6.24	**2.08**	(1.11, 3.56)	≤5	36	6.22	**5.78**	(4.05-8.00)
	≥50	59	30.57	**1.93**	(1.47, 2.49)	≤1	7	0.74	**9.42**	(3.77-19.40)
						1-5	29	5.48	**5.29**	(3.54-7.59)
						5-10	24	9.73	**2.47**	(1.58-3.67)
						≥10	12	20.85	0.58	(0.30-1.01)
Breast (174)	<50	34	29.38	1.16	(0.80, 1.62)	≤5	52	13.71	**3.79**	(2.83-4.97)
	≥50	61	37.13	**1.64**	(1.26, 2.11)	≤1	12	1.40	**8.56**	(4.42-14.96)
						1-5	42	12.31	**3.25**	(2.32-4.43)
						5-10	21	20.05	1.05	(0.65-1.60)
						≥10	22	32.75	0.67	(0.42-1.02)
Ovary (183)	<50	23	3.68	**6.25**	(3.96, 9.38)	≤5	37	1.84	**20.12**	(14.16-27.73)
	≥50	17	5.97	**2.85**	(1.66, 4.56)	≤1	30	0.20	**152.87**	(103.12-218.25)
						1-5	7	1.64	**4.26**	(1.71-8.78)
						5-10	1	2.78	0.36	(0.00-2.00)
						≥10	2	5.03	0.40	(0.04-1.44)
Vagina (184)	<50	6	0.38	**15.68**	(5.73, 34.14)	≤5	7	0.29	**24.43**	(9.79-50.34)
	≥50	10	1.38	**7.22**	(3.46, 13.29)	≤1	6	0.04	**172.12**	(62.85-374.65)
						1-5	1	0.25	**3.97**	(0.05-22.11)
						5-10	3	0.46	**6.58**	(1.32-19.23)
						≥10	6	1.02	**5.86**	(2.14-12.75)
Bladder (188)	<50	5	1.07	**4.69**	(1.51, 10.95)	≤5	7	1.22	**5.74**	(2.30-11.82)
	≥50	13	6.72	**1.93**	(1.03, 3.31)	≤1	3	0.15	**19.69**	(3.96-57.52)
						1-5	4	1.07	**3.75**	(1.01-9.59)
						5-10	4	1.99	2.01	(0.54-5.14)
						≥10	7	4.58	1.53	(0.61-3.15)
Kidney (189)	<50	4	1.68	2.38	(0.64, 6.10)	≤5	7	1.82	**3.86**	(1.54-7.95)
	≥50	21	9.47	**2.22**	(1.37, 3.39)	≤1	6	0.22	**27.70**	(10.12-60.30)
						1-5	1	1.60	330.63	(0.01-3.48)
						5-10	9	2.93	**3.07**	(1.40-5.82)
						≥10	9	6.40	1.41	(0.64-2.67)
Total	<50	128	52.37	**2.44**	(2.04, 2.91)	≤5	210	34.33	**6.12**	(5.32,7.00)
	≥50	269	135.77	**1.98**	(1.75, 2.23)	≤1	76	3.84	**19.80**	(15.60-24.75)
						1-5	134	30.50	**4.39**	(3.68-5.21)
						5-10	94	52.37	**1.79**	(1.45, 2.20)
						≥10	93	101.45	0.92	(0.74, 1.12

Bold indicates statistical significance

*SIR* standardized incidence ratio, *SPM* second primary malignancy, *O* observed numbers of SPMs, *E* expected numbers of SPMs, *CI* confidence interval

^a^Age at diagnosis of uterine cancer

^b^Follow-up interval after the diagnosis of uterine cancer

### Risk of second primary malignancies stratified by follow-up interval

SIRs stratified according to the follow-up interval from the initial diagnosis of uterine cancer were examined for the eight sites with an elevated risk of SPM (Table 3, right half). The follow-up period was divided into 4 intervals: ≤1, 1–5, 5–10, and ≥10 years. Overall, SIR was found to markedly decrease over time. The increased risk of SPM at all selected sites was the greatest in the first year and diminished with time (SIR = 19.80, 4.39, 1.79, and 0.92 for ≤1, 1–5, 5–10, and ≥10 years follow-up, respectively, P < 0.001). Furthermore, an increased risk of breast, ovarian, and bladder SPM was observed in only the first 5 years, whereas an increased risk of small intestinal, colorectal, lung, and kidney SPM remained after up to 10 years of follow-up. The vagina/vulva was the only location with a markedly increased risk of SPM, lasting for ≥10 years of follow-up.

### Cumulative incidence rates of all second cancers

**Fig. 1:**
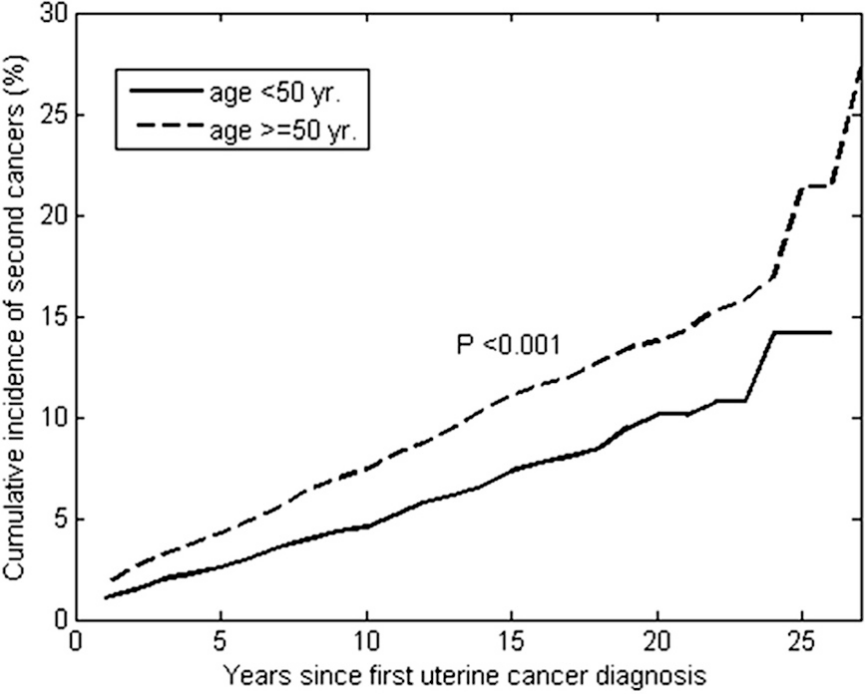
Cumulative incidence rates of all the second cancers after uterine cancer stratified by age

The estimated overall risk of developing SPM in uterine cancer survivors was calculated, with death treated as a competing risk. For all SPMs, the cumulative risk in the younger group (<50 years) at 5, 10, 15, 20, and 25 years after uterine cancer was estimated to be 1.96 %, 3.85 %, 6.47 %, 9.15 %, and 13.24 %, respectively (Fig. 1). In contrast, the cumulative incidence was higher in older patients (≥50 years) than in younger patients (3.42 %, 6.42 %, 10.07 %, 12.84 %, and 20.51 % at 5, 10, 15, 20, and 25 years, respectively). A significant difference between the two cumulative incidence curves indicated that the risk of all SPMs differed between age groups (P < 0.001; Fig. 1).

### Overall survival and impact of second primary malignancies in patients with uterine cancer

**Fig. 2:**
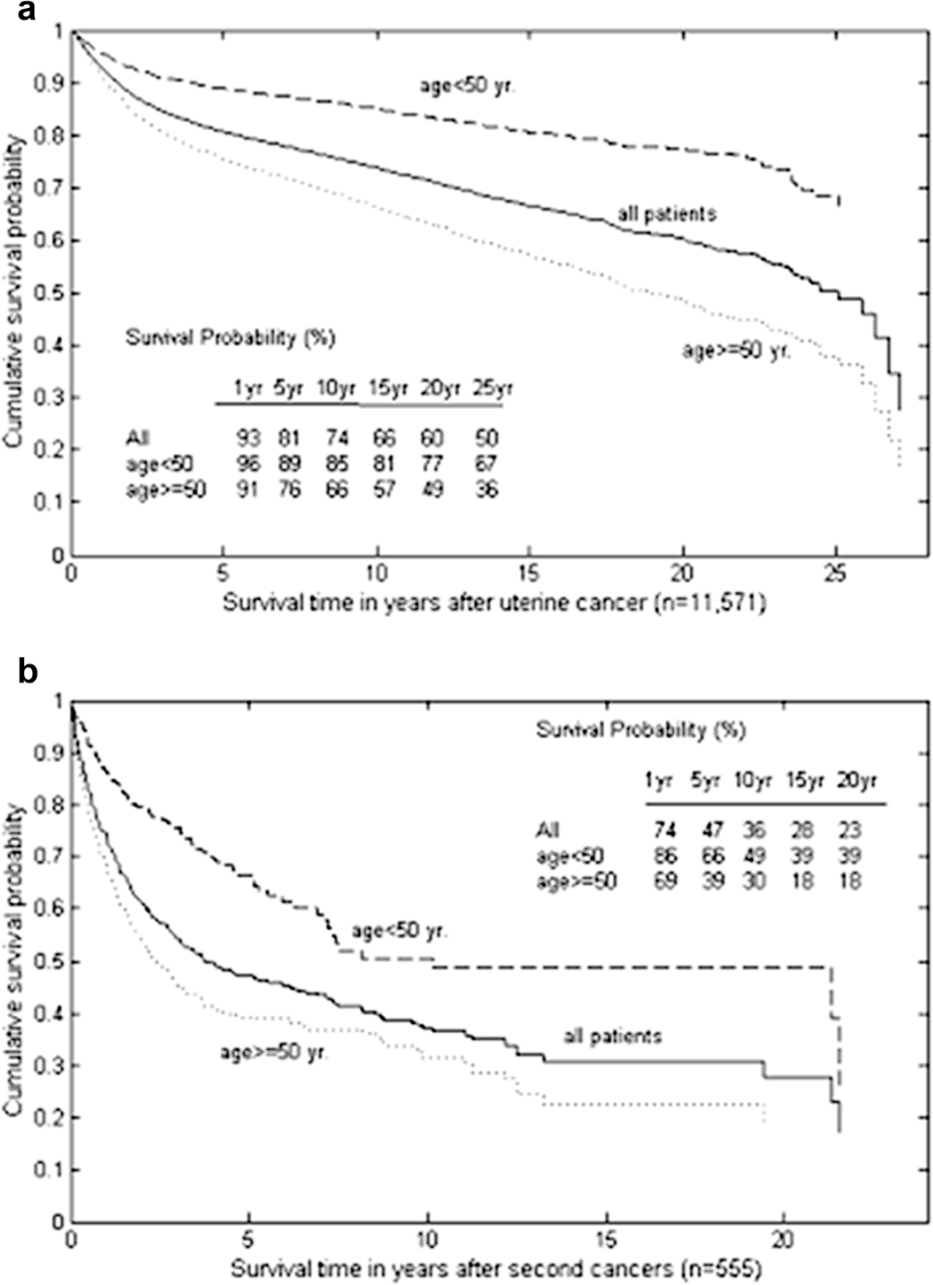
(**a**): Survival of all the patients with uterine cancer stratified by age at diagnosis of uterine cancer; (**b**): survival after second primary cancers stratified by age

The median overall survival was 25.08 years, and the five-year survival rate was 81 % (Fig. 2a). The patients diagnosed with uterine cancer at an age <50 years had a higher survival than those diagnosed at a later age (Fig. 2a, P < 0.001). For the 555 patients with SPMs, the median survival time after SPM diagnosis was 3.77 years (Fig. 2b), with 1–, 5–, 10–, and 15-year survival rates of 74 %, 47 %, 36 % and 28 %, respectively. Of these patients, the younger age group had a higher survival after diagnosis of any SPM (median survival time, 10.14 vs. 2.40 years; P < 0.001).

The Cox model with a time-dependent covariate was employed for assessing the impact of SPMs on survival (Table 4). After adjusting for age at diagnosis of uterine cancer, we found that the SPM development was associated with a markedly increased risk of death (adjusted hazard ratio = 1.74, P < 0.001). This result suggests older patients (≥50 years) at initial uterine cancer diagnosis who subsequently develop SPM are at the highest risk of death.

**Table 4:** Cox regression analysis of overall survival, with second cancer as a time-dependent covariate

		Hazard ratio	P-value
Age diagnosis age <50		1	<0.001
	≥50	2.457	
Second cancers without		1	<0.001
	with	1.771	

## Discussion

Patients in the present study had a median overall survival of 25.08 years, consistent with the reported survival of patients in the SEER database. In our study, patients with SPMs had a median survival of 3.77 years following SPM diagnosis, and the average interval between the first and second cancers was 6.27 years (SD = 5.53 years). The highest risk of developing SPM was observed in the first 5 years after diagnosis of uterine cancer, largely because of an extremely increased risk in the first year (Table 3). The close follow-up during the first year following the diagnosis may have contributed to an earlier detection of second malignancies, leading to a surveillance bias. Moreover, recommendations regarding screening should consider this observed latency pattern. Screening and preventive strategies for SPMs are essential for reducing mortality rates. However, no universal guidelines for SPM surveillance in patients with uterine cancer have been established to date. A major obstacle has been contradictory results from studies examining SPMs. For example, a nationwide Swedish population-based study reported a 54 % increased risk for all SPMs (SIR = 1.54; 95 % CI, 1.48–1.61) in 19,128 patients with primary endometrial cancer, revealing an increased risk of subsequent malignancy at 11 sites, including the ovary, urinary bladder, small intestine, connective tissue, colon or rectum, female genitalia, kidney, skin (squamous cell), breast, and bone marrow (particularly, leukemia). However, the strong decline in SPM development with the length of follow-up found in our study was not observed in the Swedish data [13]. A complete evaluation of the US SEER data revealed no increase in the overall risk of SPMs (SIR = 0.99, excluding female genital sites; 95 % CI, 0.97–1.01). However, our study corroborated findings of the SEER database regarding the association between age at onset and risk of SPM. Further, despite SIRs being generally lower in the SEER database than those reported in Taiwan, the same SPM sites were found to have increased SIRs [19]. Another study using the SEER database also found no overall increased risk of SPMs (SIR = 0.85; 95 % CI, 0.84–0.87) and reported no increased risk of breast, ovarian, or colorectal SPM [18]. In our study, there was a 71 % increased risk of SPM following uterine cancer (SIR = 1.71; 95 % CI, 1.57–1.86), with a higher risk of small intestinal, colorectal, ovarian, vaginal/vulvar, breast, urinary bladder, kidney, and lung cancers. In general, treatments for uterine cancer in Taiwan are similar to those used in Western countries, particularly the US, as Taiwanese clinicians generally follow American guidelines. Hence, the discrepancies in results in this study and those using the US SEER data may be because of follow-up time, surveillance bias, and methodology rather than differences in treatment regimes. First, the follow-up period in our study (69,987 person-years) was shorter than that in the US (705,002 person-years) and Swedish (255,211 person-years) studies [13, 19]. Second, a close follow-up in the first 5 years, particularly the first year, may have contributed to an earlier detection of SPM and lead to surveillance bias. Last, differences may have been because of the study methodology rather than intrinsic differences between the two countries. For example, the influence of migration on Taiwan is minimal compared with that on the US. The population in Taiwan is relatively stable, and mostly, Taiwanese emigrants return to Taiwan for medical treatments as the National Health Insurance Plan in Taiwan provides great medical care at a lower cost.

The mechanisms underlying SPM development are unclear; however, an association between hereditary factors, common environmental risk factors, and effects of treatment modalities between the first and second malignancy may be responsible. In the present study, the risk of SPMs in the small intestine, colon or rectum, ovary, and urinary bladder was found to be higher in patients with a younger age (<50 years) at the initial diagnosis of uterine cancer, suggesting an underlying genetic association. The discovery of metachronous cancers in a young patient suggests a hereditary cause. The most well-known example is LS, an autosomal dominant disease caused by germ-line mutations in DNA mismatch repair genes [27, 28]. LS is associated with an increased risk of colorectal, endometrial, and ovarian cancers, with the risk of endometrial cancer equaling or exceeding that of colorectal cancer. Diagnosis of gynecological cancer precedes colorectal cancer in over half of the women with LS. It has been recently reported that women with LS diagnosed with a primary endometrial cancer have an increased risk of SPMs, including colorectal, kidney, renal pelvic, ureteral, urinary bladder, ovarian, and breast cancers [29, 30]. In addition to LS, patients with genetic polymorphisms in DNA repair enzyme genes, such as ERCC1 and XPF, have also been reported to be at an increased risk of developing multiple cancers [31, 32].

The incidence of estrogen-related cancers, including that of the breast and ovary and endometrial carcinomas of the uterus, is rapidly increasing in Taiwan [4]. Although the underlying mechanisms remain unelucidated, they are believed to involve a complex association between genetic, endocrinal, and environmental factors. Xenoestrogens are widely dispersed into the environment because of increasing industrialization. Marked exposure levels of nonylphenol, ubiquitously found in water supplies and food, and bisphenol A, found in considerable amounts in polycarbonate plastics, can be detected in the Taiwanese population. The average daily intake of nonylphenol in Taiwan has been reported to be 4–and 8.5-folds higher than that in Germany and New Zealand, respectively [33]. These organic compounds, which have estrogenic effects and can cause precocious puberty and early menarche [34], resulting in an increased cumulative life-long exposure to estrogen, have been implicated in carcinogenesis [35].

Regarding SPM sites, we found that the organs within and closer to irradiated fields (vagina/vulva, small intestine, ovary, kidney, bladder, ureter, and colorectum) were at a higher risk of SPM than those not directly exposed to radiation (lung and breast). Pelvic radiotherapy for endometrial cancer is associated with a marked increase in the risk of SPM in the urinary system (kidney, ureter, bladder, and urethra), colon and rectum, vagina/vulva, sarcoma, breast, and lung [14, 16]. Radiation has also been reported to induce local immunosuppression which may activate high-risk human papillomavirus infection and increase the risk of vaginal and vulvar cancer [36, 37]. Beyond the radiation field, radiotherapy may increase the risk of lung and breast cancers through the bystander effect, in which the non-exposed cells receive signals from irradiated cells (radiation-induced genomic instability) and confer predisposition to malignancy [38]. We found that the risk of developing these SPMs was the highest within the first five years after diagnosis of uterine cancer. Considering the long latency required for carcinogenesis, the higher risk of SPMs occurring within a short period after the first cancer contradicts the hypothesis that radiotherapy is the sole SPM initiator. Factors other than radiotherapy, such as heritable factors, may influence the risk of SPM. Further, a higher than expected incidence of the second cancer, strongly associating with radiation and occurring in relatively short latency periods, has been reported in numerous studies about uterine corpus cancer [13, 16, 19]. However, this was not observed in our study.

The major limitation of this study is the lack of information regarding potential confounders. For example, data on the received treatments, staging at initial diagnosis, and treatment-related complications were not included. Information regarding bilateral oophorectomy is known to influence the estimated risk of second ovarian cancer, particularly in young patients with early stage diseases who may have undergone hysterectomy with ovary preservation or those who may undergo hysterectomy without oophorectomy for presumed uterine myoma or adenomyosis prior a histological diagnosis of malignancy. Hence, the calculated SIR of second ovarian cancer may have been underestimated. Moreover, SIRs in the first follow-up year were extremely high for second ovarian and vaginal/vulvar cancers (SIR = 152.87; 95 % CI, 103.12–218.25 and SIR = 172.12; 95 % CI, 62.85–374.65, respectively Table 3). This may have been because of a high prevalence of synchronous malignancies involving anatomically related organs as 22 of 30 s ovarian and 4 of 6 s vaginal/vulvar cancers occurred within 6 months of diagnosis of uterine cancer.

## Conclusions

The present study suggests the higher incidence of SPM in women previously diagnosed with uterine cancer may be because of genetic, environmental, and therapy-related factors. The overall risks and patterns of SPMs in Taiwan differ from those reported in the United States suggesting the relative influence of each factor differs according to the patient demographics, study methodology, and follow-up period. Therefore, surveillance guidelines for SPMs should be developed using data specific to local populations.

## Abbreviations

CI: Confidence interval
E: Expected number
IACR: International Association of Cancer Registries
IARC: International Agency for Research on Cancer
ICD-9: International Classification of Diseases, 9th Revision
O: Observed number
LS: Lynch syndrome
SEER: Surveillance, Epidemiology, and End Results
SIR: Standardized incidence ratio
SPM: Second primary malignancy
TCR: Taiwan Cancer Registry
